# Early post-TAVI thrombocytopenia recovery and subsequent subclinical leaflet thrombosis

**DOI:** 10.1093/ehjopen/oeag011

**Published:** 2026-01-30

**Authors:** Marco Moscarelli, Thanos Athanasiou, Roberto Casula, Mai-Linh Nguyen Trung, Petitjean Hélène, Oury Cecile, Massimo Salardino, Vincenzo Pernice, Gregorio Zaccone, Sabrina Milo, Giuseppe Speziale, Khalil Fattouch, Patrizio Lancellotti

**Affiliations:** Department of Cardiovascular Surgery, Maria Eleonora Hospital, Viale Regione Siciliana Nord Ovest 1571, 90135 Palermo, Italy; Department of Surgery and Cancer, Hammersmith Hospital, Du Cane Road, London W12 0NN, UK; Department of Surgery and Cancer, Hammersmith Hospital, Du Cane Road, London W12 0NN, UK; Department of Cardiac Surgery, Hammersmith Hospital, Du Cane Road, London W12 0HS, UK; Department of Surgery and Cancer, Hammersmith Hospital, Du Cane Road, London W12 0NN, UK; Department of Cardiac Surgery, Hammersmith Hospital, Du Cane Road, London W12 0HS, UK; GIGA Institutes, Cardiovascular Sciences and Metabolism, Department of Cardiology, CHU – B34 Quartier Hôpital, Avenue de l'Hôpital, 11 4000 Liège, Belgium; GIGA Institutes, Cardiovascular Sciences and Metabolism, Department of Cardiology, CHU – B34 Quartier Hôpital, Avenue de l'Hôpital, 11 4000 Liège, Belgium; GIGA Institutes, Cardiovascular Sciences and Metabolism, Department of Cardiology, CHU – B34 Quartier Hôpital, Avenue de l'Hôpital, 11 4000 Liège, Belgium; Department of Cardiovascular Surgery, Maria Eleonora Hospital, Viale Regione Siciliana Nord Ovest 1571, 90135 Palermo, Italy; Department of Cardiovascular Surgery, Maria Eleonora Hospital, Viale Regione Siciliana Nord Ovest 1571, 90135 Palermo, Italy; Department of Cardiovascular Surgery, Maria Eleonora Hospital, Viale Regione Siciliana Nord Ovest 1571, 90135 Palermo, Italy; Department of Radiology, Maria Eleonora Hospital, Viale Regione Siciliana Nord Ovest 1571, 90135 Palermo, Italy; Department of Cardiovascular Surgery, Santa Maria Hospital, Via Antonio De Ferrariis 22, 70124 Bari, Italy; Kore University of Medicine, Piazza della Università, 94100 Enna, Italy; GIGA Institutes, Cardiovascular Sciences and Metabolism, Department of Cardiology, CHU – B34 Quartier Hôpital, Avenue de l'Hôpital, 11 4000 Liège, Belgium

**Keywords:** TAVI, Subclinical leaflet thrombosis, Thrombocytopenia, TAVR, Transcatheter aortic valve implantation

## Abstract

**Objective:**

Post-implant thrombocytopenia and subclinical leaflet thrombosis (SLT) are two common phenomena, yet their potential interplay remains unexplored. This study aimed to determine whether early platelet dynamics differed in patients diagnosed with SLT on multidetector computed tomography (MDCT) compared with those without SLT.

**Methods and results:**

A total of 118 consecutive patients treated with self-expandable supra-annular and intra-annular prostheses were longitudinally analysed. Platelet count was assessed using the corrected platelet count (CPC) from baseline to day 7 (or hospital discharge) and at 30 days. Platelet-to-lymphocyte ratio (PLR) and lymphocyte-to-monocyte ratio (LMR) were also measured as inflammatory markers. Spleen volume was assessed at baseline. MDCT was performed at 6-month follow-up to evaluate SLT. SLT, ranging from mild to severe, was detected in 22 patients at the 6-month MDCT follow-up. The SLT group had a lower baseline platelet count compared to the No-SLT group, with persistently lower levels from day 1 to day 7 post-implant. Platelet count was significantly lower in the SLT group on days 5 and 6 (CPC: 170 (IQR: 50) × 10³/μL vs. 215 (IQR: 72) × 10³/μL, *P* = 0.048; and 175 (IQR: 55) × 10³/μL vs. 240 (IQR: 85) × 10³/μL, *P* = 0.038). No significant differences were observed in PLR or LMR. Spleen volume was also significantly lower in the SLT group.

**Conclusion:**

We demonstrated a reversible thrombocytopenia during the first week post-implant, which was more pronounced in the SLT group than in the No-SLT group. Additionally, spleen volume was smaller in the SLT group, suggesting a potential interplay between these factors.

## Graphical Abstract

**Graphical Abstract:**
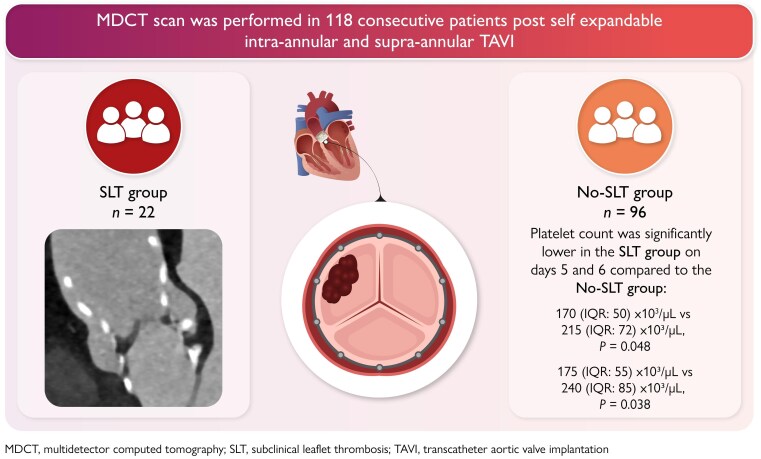
Early Post-TAVI Thrombocytopenia Recovery and Subsequent Subclinical Leaflet Thrombosis.

## Introduction

Post-implantation thrombocytopenia and subclinical leaflet thrombosis (SLT), also referred to as hypoattenuated leaflet thickening (HALT), are two common findings following transcatheter aortic valve implantation (TAVI),^1,2^. Post-TAVI thrombocytopenia typically occurs early after the procedure and is generally transient, yet it has been associated with worse short- and mid-term outcomes in some studies.^3^ In parallel, SLT/HALT has emerged as an imaging-defined phenomenon detected by multidetector computed tomography (MDCT), and may represent an early manifestation of structural valve alteration, with potential clinical implications.^1^

Despite their frequent coexistence in the post-TAVI setting, the relationship between early platelet dynamics and subsequent SLT/HALT remains poorly defined. Most prior studies have evaluated thrombocytopenia as a static event, rather than focusing on the temporal pattern of platelet recovery during the immediate post-procedural period.^3^ Similarly, investigations of SLT/HALT have largely focused on anatomical and procedural determinants, with limited attention to early haematological changes.

It remains uncertain whether differences in early post-TAVI platelet recovery are associated with the later detection of SLT/HALT. Clarifying this relationship may help to improve the understanding of early post-implant biological responses and their potential link to subsequent valve findings.

Therefore, the present study aimed to

- characterize the incidence and severity of early post-TAVI thrombocytopenia according to the presence or absence of SLT/HALT;
- evaluate platelet recovery trajectories during the early post-procedural period; and
- explore inflammatory haematological indices, including the platelet-to-lymphocyte ratio (PLR) and lymphocyte-to-monocyte ratio (LMR).

Baseline indexed spleen volume was additionally assessed as a hypothesis-generating variable given its potential association with platelet dynamics.

## Patients and methods

### Study population

**Figure 1:**
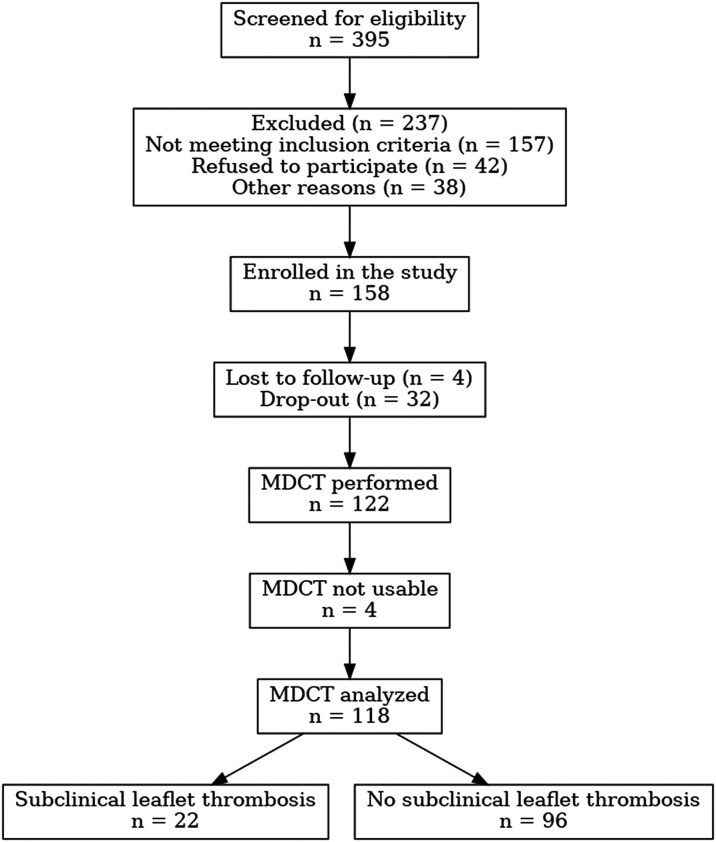
Study flow chart. Flow diagram illustrating patient selection for the present analysis, including inclusion criteria and final study population stratified by the presence or absence of subclinical leaflet thrombosis (SLT) at 6-month multidetector computed tomography follow-up.

This study was conducted within the EndoTAVI Project, a European grant-funded initiative (PO-FESR 2014–2020; www.endotavi.it) aimed at identifying predictors of early valve-related alterations after transcatheter aortic valve implantation (TAVI). Consecutive patients undergoing TAVI for symptomatic severe aortic stenosis at a tertiary university centre between January 2019 and July 2024 were considered (*Figure 1*).

Patients were included if complete platelet count data were available at baseline and during the early post-procedural period, and if contrast-enhanced multidetector computed tomography (MDCT) suitable for the assessment of subclinical leaflet thrombosis (SLT) was available at 6-month follow-up. Baseline thrombocytopenia was not an exclusion criterion. The study population selection process is summarized in a flow chart.

The study was conducted in accordance with the Declaration of Helsinki and STROBE guidelines and was approved by the local ethics committee.^4^ All patients provided written informed consent.

### Platelet measurements and laboratory variables

Blood samples were collected at baseline (pre-procedural), daily during the first 7 days after TAVI or until discharge, and at 30-day follow-up. Platelet count was analysed using the corrected platelet count (CPC), calculated by adjusting platelet values for peri-procedural haemodilution based on changes in haematocrit.^5^ CPC was evaluated both as absolute values and as longitudinal trajectories over time. Thrombocytopenia severity was classified according to standard platelet count thresholds, and platelet recovery was assessed by examining the temporal pattern of CPC normalization during early follow-up.

Inflammatory haematological indices, including the platelet-to-lymphocyte ratio (PLR) and lymphocyte-to-monocyte ratio (LMR), were calculated using contemporaneous complete blood count measurements and analysed as exploratory variables.^6^

### MDCT acquisition and SLT definition

All patients underwent contrast-enhanced, electrocardiogram-gated MDCT at 6 months after TAVI using standardized acquisition protocols. SLT was defined as the presence of hypoattenuated leaflet thickening with or without associated restriction of leaflet motion, according to established criteria.^7^ Image analysis was performed by experienced readers blinded to clinical and laboratory data.

### Splenic volume assessment

Baseline splenic volume was assessed on pre-procedural MDCT using volumetric reconstruction and indexed to body surface area.^8^ This parameter was analysed as a hypothesis-generating variable due to its potential association with platelet dynamics.

### Statistical analysis

Continuous variables are reported as mean ± standard deviation or median (interquartile range), and categorical variables as counts and percentages. Group comparisons were performed using appropriate parametric or non-parametric tests.

Longitudinal platelet count trajectories were analysed using linear mixed-effects models with random intercepts to account for repeated measurements within individuals. Fixed effects included time, SLT status, and their interaction to assess differences in platelet trends over time between groups. A two-sided *P* value <0.05 was considered statistically significant. Statistical analyses were performed using R software.

## Results

### Study population and baseline characteristics

Among 118 consecutive patients undergoing TAVI, SLT was identified on 6-month multidetector computed tomography in 22 patients (18.6%). Baseline clinical and procedural characteristics of the overall cohort and stratified by SLT status are summarized in *Table 1*. No significant differences were observed between groups with respect to baseline echocardiographic parameters, valve platform, or procedural characteristics. Antithrombotic therapy before implantation and at the time of follow-up imaging was comparable between the SLT and no-SLT groups.

**Table 1:** Characteristics of the patients at baseline, peri-procedural details, burden of thrombosis

	Overall cohort N = 118	SLT		
		NO N = 96	YES N = 22	*P*-value
Age, yr	78.4 (5.7)	78.1 (5.7)	79.6 (5.8)	0.263
BSA^a^, m^2^	1.8 (0.2)	1.8 (0.2)	1.8 (0.2)	0.737
BMI^b^	28.1 (5.0)	27.8 (4.5)	29.3 (6.8)	0.200
BMI≥30	31 (26.5)	24 (25.3)	7 (31.8)	0.719
Male sex, no. (%)	62 (52.5)	53 (55.2)	9 (40.9)	0.330
Euroscore II, %	1.8 (1.8)	1.8 (1.7)	1.8 (1.7)	0.222
Low flow—low gradient, n (%)	12 (11.5)	9 (10.5)	3 (16.7)	0.731
NYHA functional class III–IV, no. (%)	14 (13.9)	14 (16.9)	0	0.133
NIDDM/IDDM, no. (%)	37 (31.9)	30 (31.6)	7 (33.3)	0.999
Hypertension, no. (%)	114 (98.3)	94 (98.9)	20 (95.2)	0.798
COPD, no. (%)	19 (17.8)	15 (16.9)	4 (22.2)	0.837
Previous stroke/TIA, no. (%)	6 (5.6)	6 (6.7)	0	0.567
Previous PCI, no. (%)	24 (22.4)	21 (23.6)	3 (16.7)	0.739
Previous cardiac surgery, no. (%)	10 (9.4)	8 (9.1)	2 (11.1)	0.988
Previous MI, no. (%)	20 (18.7)	17 (19.1)	3 (16.7)	0.997
Coronary artery disease, no. (%)	30 (28.3)	25 (28.4)	5 (27.8)	0.776
Serum creatinine, mg/dL	0.8 (0.2)	0.8 (0.4)	0.8 (0.7)	0.659
Creatinine clearance by Cockcroft–Gault formula (mL/min)	67 (48.1)	67 (37.2)	68.2 (49.5)	0.713
Pre-existing pacemaker or defibrillator, no. (%)	11 (11.0)	7 (8)	4 (22.2)	0.166
History of right bundle-branch block, no. (%)	3 (3.0)	2 (2.4)	1 (5.6)	0.999
*Procedural characteristics:*				0.422
Valve type				
Evolut R	105 (89.0)	86 (89.6)	19 (86.4)	
Portico	8 (6.8)	7 (7.3)	1 (4.5)	
Navitor	5 (4.2)	3 (3.1)	2 (9.1)	
Valve size				0.582
23 mm	10 (8.5)	9 (9.5)	1 (4.5)	
25 mm	3 (2.5)	2 (2.1)	1 (4.5)	
26 mm	45 (38.5)	37 (38.9)	8 (36.4)	
27 mm	5 (4.3)	3 (3.2)	2 (9.1)	
29 mm	39 (33.3)	31 (32.6)	8 (36.4)	
34 mm	16 (13.7)	14 (14.7)	2 (9.1)	
Pre-TAVR balloon valvuloplasty	37 (31.4)	32 (33.3)	5 (22.7)	0.476
Post-TAVR balloon valvuloplasty	51 (43.2)	41 (42.7)	10 (45.5)	0.989
**Burden of subclinical leaflet thrombosis and restricted leaflet movement**				
**Patient with leaflet thrombosis**, n (%)^c^	22 (18.6)			
Involvement of a single leaflet	10 (8.4)			
Involvement of two leaflets	5 (4.2)			
Involvement of three leaflets	7 (5.9)			
**Hypoattenuated lesions leaflet n (%)**				
HALT ≤ 25%	10 (8.4)			
HALT > 25%—50%	21 (17.7)			
HALT > 50%—75%	2 (1.7)			
HALT > 75%	8 (6.7)			
**Restricted leaflet movement (%)**				
RELM ≤ 25%	19 (16.1)			
RELM > 25%—50%	20 (16.9)			
RELM > 50%—75%	2 (1.69)			
RELM > 75%	0			

Values are reported as the mean and standard deviation (SD) or number and percentage (%). BMI: body mass index. BSA: body surface area. COPD: chronic obstructive pulmonary disease. HALT: Hypoattenuated leaflet thickening. IDDM: insulin-dependent diabetes mellitus. MI: myocardial infarction. NIDDM: non-insulin-dependent diabetes mellitus. NYHA: New York Heart Association. PCI: percutaneous coronary intervention. RELM: Restricted leaflet movement. SLT: Subclinical leaflet thrombosis. TIA: transient ischaemic attack.

^a^Body surface area was calculated with the Du Bois formula: 0.007 × weight^0.425^ × height^0.725^.

^b^The body mass index was calculated as the weight in kilograms divided by the square of the height in metres.

Data were available for the entire cohort. ^c^One patient may have more than one of SLT/HALT simultaneously

### Early platelet dynamics and recovery

**Figure 2:**
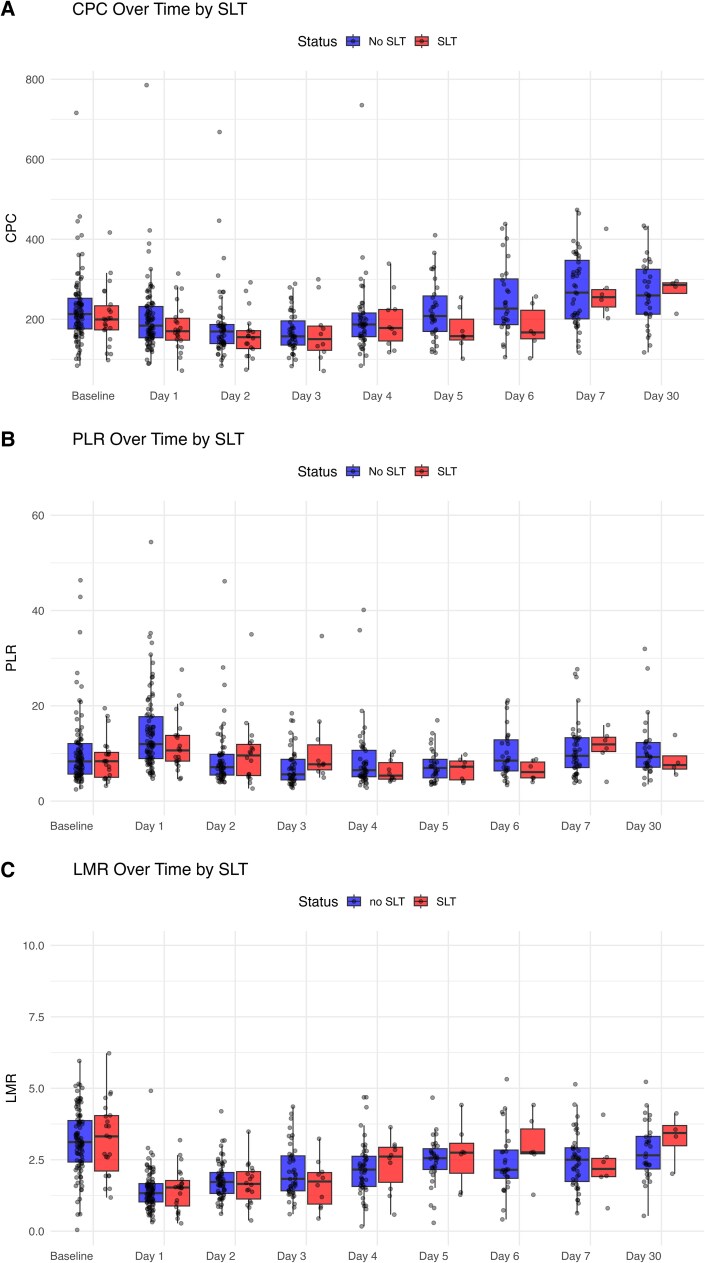
Early platelet dynamics and recovery after TAVI stratified by SLT status. Longitudinal trajectories of corrected platelet count (CPC) during the early post-procedural period in patients with and without subsequent subclinical leaflet thrombosis (SLT). Platelet recovery was delayed in patients who developed SLT. CPC: corrected platelet count; SLT: subclinical leaflet thrombosis.

Baseline platelet count was numerically lower in patients who subsequently developed SLT, although this difference was not statistically significant. Both groups experienced a significant early decline in platelet count following TAVI, with a nadir occurring within the first 2–3 post-procedural days (mixed-effects model for time: *P* < 0.001) (*Figure 2A–C*).

While overall platelet trajectories did not differ significantly between groups (time × SLT interaction *P* = 0.86), platelet recovery during the early post-procedural phase was delayed in patients with SLT. Platelet counts were significantly lower in the SLT group on post-procedural days 5 and 6 compared with the No-SLT group (*Figure 2*). A subsequent recovery phase was observed in both groups.

### Severity of thrombocytopenia

At least moderate thrombocytopenia (<100 × 10³/μL) occurred more frequently in the SLT group during the early post-procedural period, with the highest incidence observed on post-procedural day 2. Severe thrombocytopenia (<50 × 10³/μL) was rare and occurred in only one patient, who belonged to the SLT group.

### Inflammatory haematological indices

Both platelet-to-lymphocyte ratio (PLR) and lymphocyte-to-monocyte ratio (LMR) showed significant temporal variation following TAVI (*P* < 0.001 for time); however, their trajectories did not differ between SLT and No-SLT groups (interaction *P* > 0.7 for both). No association was observed between these inflammatory indices and subsequent SLT.

### Indexed spleen volume

Baseline indexed spleen volume was significantly smaller in patients with SLT compared with those without SLT. Splenomegaly was more frequently observed in the No-SLT group. Given the exploratory nature of this analysis, spleen volume findings were considered hypothesis-generating.

## Discussion

In this report, we describe an association between early post-procedural platelet recovery patterns and the subsequent detection of subclinical leaflet thrombosis (SLT) following transcatheter aortic valve implantation (TAVI). While thrombocytopenia and SLT/HALT are both common findings after TAVI, their temporal relationship has not been well characterized. Our data suggest that patients who later develop SLT experience a more pronounced and delayed platelet recovery during the first post-implantation week.

Importantly, this difference was observed in the recovery phase rather than in the absolute platelet nadir, and baseline platelet counts were not significantly different between groups. This finding supports the concept that early platelet dynamics, rather than isolated platelet values, may be relevant in the post-TAVI setting. Both groups exhibited a transient early platelet decline, consistent with prior reports, followed by gradual recovery.

SLT/HALT has been increasingly recognized as an early post-implant imaging phenomenon, with previous MDCT studies demonstrating that leaflet thrombosis may develop within weeks to months after TAVI. Our findings suggest that early haematological changes precede the detection of SLT, although the observational nature of this study precludes any causal inference.

Baseline indexed spleen volume was smaller in patients with SLT. Given the exploratory nature of this analysis and the limited sample size, this observation should be considered hypothesis-generating and interpreted with caution. The potential relationship between splenic platelet handling and post-TAVI platelet dynamics warrants further investigation in dedicated prospective studies.

Inflammatory haematological indices, including platelet-to-lymphocyte and lymphocyte-to-monocyte ratios, did not differ between groups, suggesting that these routinely available markers do not capture the biological processes underlying SLT in this context.

This study has limitations. The sample size was modest, and platelet function, von Willebrand factor abnormalities, and molecular markers of platelet activation were not assessed. Imaging was limited to a single follow-up time point, and the analysis was restricted to self-expandable valve platforms. Finally, the study was not designed to develop predictive models or assess the impact of early platelet changes on clinical outcomes.

## Conclusions

Early post-TAVI platelet recovery is delayed in patients who subsequently develop subclinical leaflet thrombosis. This association highlights early platelet dynamics as a potential marker of subsequent valve-related findings. The observed relationship between platelet recovery patterns and SLT, as well as the exploratory findings related to spleen volume, warrant confirmation in larger prospective studies incorporating serial imaging and platelet function assessment.

## Lead author biography


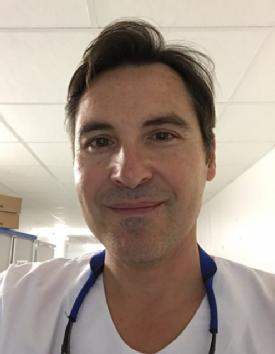


Professor Patrizio Lancellotti is Director of the Interdisciplinary Cluster for Applied Genoproteomics and Head of the Cardiology Department at the University Hospital of Liège, Belgium. He obtained his MD and PhD from the University of Liège, where he also completed his cardiology and intensive care training. A Fellow of the European Society of Cardiology (FESC), he is internationally recognized for his work in valvular heart disease, cardiovascular imaging, and cardio-oncology. Professor Lancellotti has served as President of the Belgian Society of Cardiology, Chair of the ESC Council on Valvular Heart Disease, and Associate Editor for both the *European Heart Journal* and the *European Heart Journal—Cardiovascular Imaging*. He currently serves as section editor for the *European Heart Journal* Open.

He has authored more than 330 peer-reviewed publications and several major cardiology textbooks.

## Data Availability

The datasets generated and/or analysed during the current study are available from the corresponding author upon reasonable request.
